# Corticosteroid-responsive portal lymphadenopathy mimicking malignant progression induced by furmonertinib in EGFR-mutated NSCLC: a case report

**DOI:** 10.3389/fphar.2026.1827175

**Published:** 2026-07-23

**Authors:** Nengyang Zhu, Guo Long, Jing Zhu, Weiguo Xu, Junhua Wu, Jiajun Xie

**Affiliations:** Department of Respiratory and Critical Care Medicine, Mianyang Central Hospital, School of Medicine, University of Electronic Science and Technology of China, Mianyang, China

**Keywords:** corticosteroid, DILI (drug induced liver injury), EGFR- TKI, furmonertinib, lymphadenopathy

## Abstract

**Background:**

Third-generation epidermal growth factor receptor tyrosine kinase inhibitors (EGFR-TKIs) are widely used in the treatment of NSCLC with EGFR mutations due to their efficacy and favourable safety profile. However, drug-induced liver injury (DILI) associated with targeted therapy may occasionally present with atypical clinical and radiological features, posing diagnostic challenges. Here, we present a rare case of severe hepatotoxicity accompanied by pseudo-progressive portal lymphadenopathy that occurred following furmonertinib treatment.

**Case presentation:**

A 59-year-old female patient with EGFR-mutated NSCLC initiated treatment with the third-generation EGFR-TKI furmonertinib. Two months into the therapy, the patient developed jaundice and marked elevations in total bilirubin and transaminases. Extensive laboratory investigations excluded viral hepatitis and autoimmune liver diseases. Abdominal imaging revealed newly enlarged portal lymph nodes, mimicking malignant metastasis, but without clear evidence of hepatic metastasis or biliary obstruction. Furmonertinib was discontinued and systemic corticosteroid therapy was initiated. Within 2 weeks, liver function tests had improved significantly and follow-up imaging showed that the portal lymphadenopathy had regressed. Causality assessment using the RUCAMyielded a score of 9, indicating a highly probable drug-induced liver injury.

**Conclusion:**

This case highlights that severe hepatotoxicity associated with furmonertinib may present with portal lymphadenopathy that mimics disease progression. Clinicians should remain vigilant for this atypical hepatotoxicity when using EGFR-TKIs and promptly assess portal lymph node status upon the emergence of jaundice or abnormal liver function. The implementation of early intervention strategies may facilitate recovery and help avoid misinterpretation of imaging findings.

## Introduction

EGFR mutations represent a significant oncogenic driver in NSCLC, particularly prevalent in East Asian populations, with an incidence of 40%–50% (Zhang et al., 2016). Targeted therapy with TKIs is established as the prevailing standard of care for this particular patient demographic. Furmonertinib (AST2818), a novel third-generation EGFR-TKI, has demonstrated promising efficacy and favourable central nervous system penetration compared to earlier generations. This is owing to its dual activity against both EGFR-sensitising and T790M resistance mutations (Shi et al., 2022; Soria et al., 2018).

While third-generation TKIs generally demonstrate a favourable safety profile (Zhou S. et al., 2024), DILI remains a clinically significant concern. Whilst clinical trials frequently highlight mild, transient elevations of transaminases, real-world data suggest that the risk of severe hepatotoxicity may be underestimated (EASL Clinical Practice Guidelines, 2019; Cheng et al., 2022). As demonstrated in landmark clinical trials and prospective surveillance restricted to third-generation EGFR-TKIs, such as the Phase 3 FLAURA and FURLONG studies (Shi et al., 2022; Soria et al., 2018), the baseline incidence of severe Grade 3 or higher hepatotoxicity or true idiosyncratic DILI frequently ranges from 1% to 2%. However, emerging post-marketing surveillance and retrospective cohorts indicate that in unselected routine oncology practices, this risk can be further amplified. The primary motivation for this clinical expansion is the acknowledged heterogeneity of the real-world population. This heterogeneity encompasses advanced age, subtle pre-existing hepatic comorbidities, and potential drug-drug interactions resulting from concomitant medications.

DILI presents with significant heterogeneity, which complicates the distinction between direct metabolic toxicity and immune-mediated reactions. Emerging evidence indicates that a subset of EGFR-TKI-induced liver injuries shares pathological features with autoimmune hepatitis, characterised by marked lymphocytic infiltration and high sensitivity to corticosteroid therapy (De Martin et al., 2018; Hoofnagle and Björnsson, 2019). For immune-mediated DILI phenotypes where conventional hepatoprotective drugs are ineffective, early glucocorticoid therapy is essential. Standard management involves the administration of prednisone orally or methylprednisolone intravenously, at a dose of 0.5–1.0 mg/kg/day. It is crucial that doses are gradually tapered over 4–6 weeks to prevent inflammatory rebound (Chalasani et al., 2021).

Clinically, this diagnostic dilemma is further complicated when immune-mediated hepatotoxicity presents with atypical extrahepatic findings, specifically reactive lymphadenopathy in the portal area. In the oncological context, such manifestations are susceptible to misinterpretation as disease progression (PD) or biliary obstruction secondary to nodal metastasis (Kunimasa et al., 2020). This phenomenon of drug-induced “pseudo-progression”, poses a significant clinical challenge. Misdiagnosis arising from this phenomenon can result in the premature discontinuation of effective targeted therapy or the administration of unnecessary invasive interventions.

In this paper, we present a rare instance of an EGFR-mutated NSCLC patient who exhibited severe liver injury with cholestatic features accompanied by portal lymphadenopathy during treatment with the tyrosine kinase inhibitor, furmonertinib. The clinical presentation was consistent with malignant progression, but this resolved rapidly following drug withdrawal and corticosteroid administration. The objective of this report is to elucidate the clinical characteristics of furmonertinib-associated immune-mediated hepatotoxicity and to emphasise the importance of distinguishing this “pseudo-progression” from true malignancy in order to optimise patient management.

## Case presentation

**FIGURE 1:**
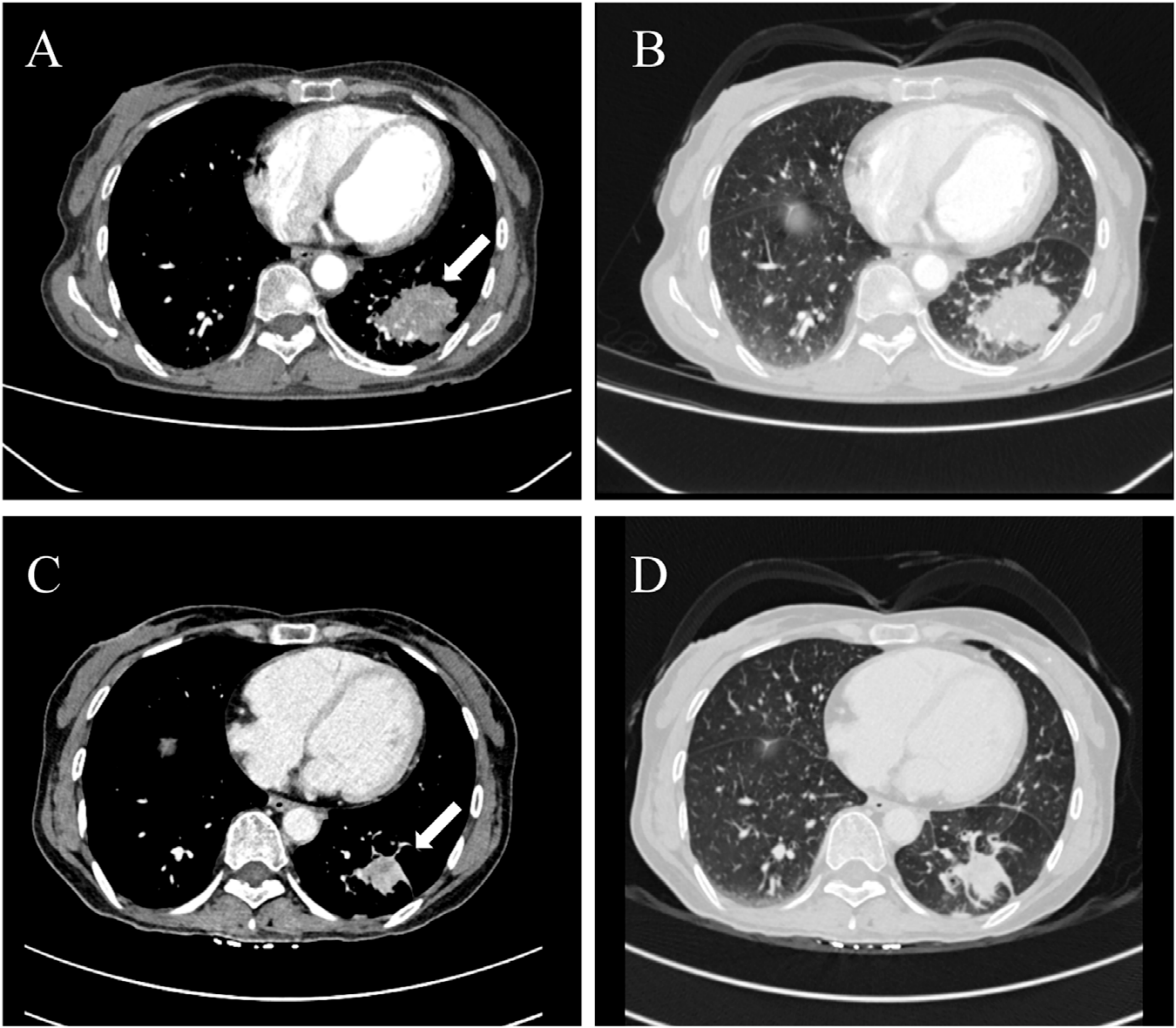
Chest CT evaluation demonstrating the therapeutic efficacy of furmonertinib on the primary lung tumor. **(A,B)** Baseline CT scans obtained on May 26, 2025. The mediastinal window **(A)** and lung window **(B)** reveal a large, solid mass in the left lower lobe (white arrow) prior to treatment. **(C,D)** Follow-up CT scans obtained on August 2, 2025, coinciding with the onset of hepatotoxicity. The mediastinal window **(C)** and lung window **(D)** demonstrate a marked reduction in tumor size (white arrow), indicating a PR to furmonertinib despite the adverse hepatic event.

**FIGURE 2:**
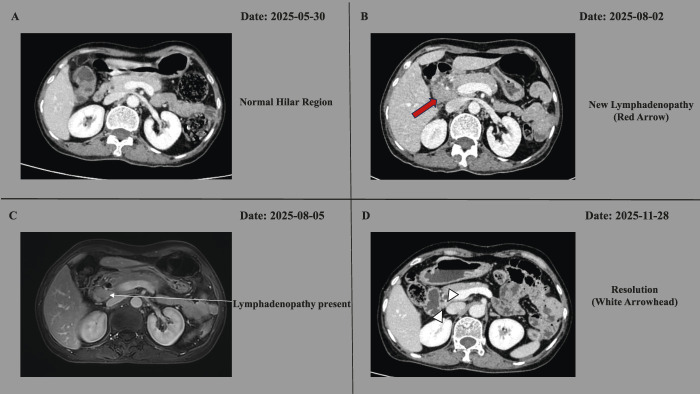
Dynamic radiological evolution of the hepatic hilar region mimicking disease progression. **(A)** Baseline contrast-enhanced CT demonstrating no enlarged no enlarged portal lymph nodes in the hepatic hilum.**(B)**CT performed at the onset of jaundice showing a newly developed portal lymphadenopathy, with the largest lymph node measuring 2.7 × 2.3 cm (red arrow). **(C)** MRI confirming lymphadenopathy, with the largest lymph node measuring 3.1 × 2.0 cm (white arrow). **(D)** Follow-up CT showing complete resolution of the previously enlarged portal lymph nodes, with no residual enlarged lymphadenopathy identified at the same anatomical location (white arrowhead).

A 59-year-old female patient with no prior history of viral hepatitis, alcohol abuse, or underlying liver disease was admitted to our department on May 25, 2025, presenting with cough and chest pain. A baseline chest CT scan revealed a solid mass in the left lower lobe (Figure 1A). Histopathological examination via percutaneous biopsy confirmed the diagnosis of NSCLC. Molecular profiling identified an EGFR exon 19 deletion mutation. Baseline abdominal imaging on May 30, 2025, showed a normal portal region with no evidence of metastasis or lymphadenopathy (Figure 2A). Notably, a history of asymptomatic gallstones was recorded.

The patient initiated targeted therapy with Furmonertinib (80 mg once daily) on June 10 2025. The treatment was initially well-tolerated. However, approximately 2 months later, on August 01, 2025, the patient presented with nausea and vomiting. Physical examination revealed distinct jaundice.

**FIGURE 3:**
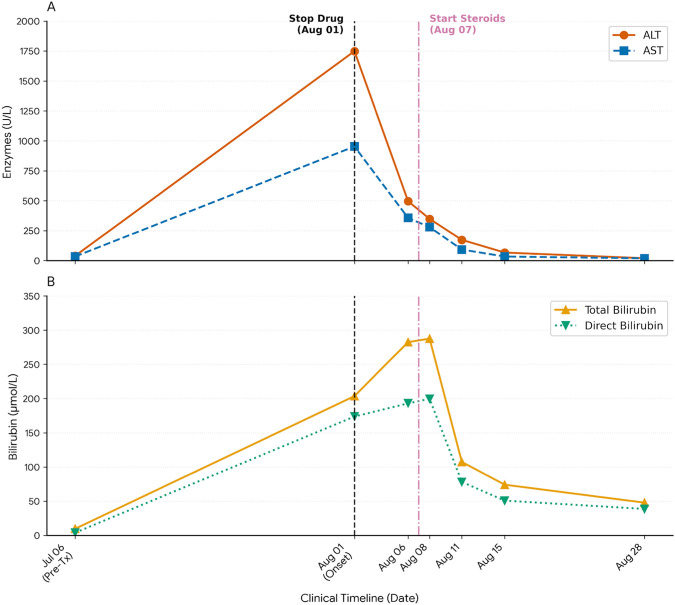
Timeline of liver function test results illustrating the clinical course of severe hepatotoxicity and the response to interventions. **(A)** Dynamic changes in hepatocellular injury markers: Alanine Aminotransferase (ALT) and Aspartate Aminotransferase (AST). Note the rapid surge peaking at the onset of symptoms (August 01) and the sharp decline following drug discontinuation and the initiation of corticosteroid therapy (August 07). **(B)** Dynamic changes in cholestasis markers: Total Bilirubin and Direct Bilirubin. The bilirubin levels continued to rise initially after drug withdrawal but showed a dramatic resolution immediately after the administration of steroids, highlighting the inflammatory nature of the cholestasis. Vertical dashed lines indicate key intervention points: cessation of furmonertinib (black) and initiation of systemic corticosteroids (pink).

Laboratory investigations indicated severe grade 3 hepatotoxicity (Figure 3A). ALT (reference range: 7–40 U/L) and AST (reference range: 13–35 U/L) levels surged to peaks of 1749 U/L and 953 U/L, respectively. TBil (reference range: 0–21.0μmol/L)was significantly elevated to 203.5 μmol/L at onset, accompanied by a DBil dominance, suggesting mixed hepatocellular and cholestatic injury.

To investigate the etiology, urgent imaging was performed. A thoracoabdominal CT on August 02, 2025, demonstrated a marked reduction in the primary tumor size compared to baseline, indicating a partial response (PR) to Furmonertinib despite the adverse event (Figure 1C). Paradoxically, the abdominal CT revealed newly developed portal lymphadenopathy, with the largest lymph node measuring 2.7 × 2.3 cm (red arrow, Figure 2B), whereas no enlarged portal lymph nodes had been identified on baseline imaging prior to treatment initiation (Figure 2A). Subsequent MRI on August 05, 2025, confirmed portal lymphadenopathy, with the largest lymph node measuring 3.1 × 2.0 cm (white arrow, Figure 2C), while excluding biliary tract obstruction, choledocholithiasis, or hepatic metastasis.

Furmonertinib was immediately discontinued on August 01, 2025. The patient was initially managed with hepatoprotective agents, including magnesium isoglycyrrhizinate, polyene phosphatidylcholine, and bicyclol. Despite drug withdrawal, cholestasis indicators continued to worsen, with TBil peaking at nearly 290 μmol/L on August 08, 2025 (Figure 3B).

Given the suspicion of immune-mediated hypersensitivity and the progressive nature of the cholestasis, systemic corticosteroid therapy was initiated on August 07, 2025, with methylprednisolone at a dose of 40 mg twice daily.

The clinical response to corticosteroids was rapid and robust. Liver enzymes declined sharply, and bilirubin levels showed a dramatic decrease immediately following steroid administration (Figure 3). The patient was discharged on August 16, 2025, with a tapering schedule of oral prednisone. A follow-up CT scan on 28 November 2025 demonstrated complete radiological resolution of the previously enlarged portal lymph nodes. No residual enlarged lymphadenopathy was identified at the same anatomical location (Figure 2D). Alongside the significant improvement in liver biochemistry following corticosteroid therapy, these results were deemed to be most consistent with pseudo-progressive inflammatory lymphadenopathy rather than malignant nodal metastasis.To evaluate the causal relationship between furmonertinib and acute liver injury systematically, the updated RUCAM scale was applied (Danan and Teschke, 2015). The assessment yielded a total score of 9, categorising the relationship as ‘highly probable’ (≥9 points). To maintain the conciseness of the main text while ensuring absolute methodological transparency, the detailed, itemized RUCAM scoring breakdown and clinical justifications based on the original Table 2 criteria have been provided in Supplementary Table S1.

## Discussion

While the Phase III FURLONG study demonstrated the safety advantage of furmonertinib, with an extremely low incidence of Grade 3 or higher hepatotoxicity (approximately 1%–2%) (Shi et al., 2022).The clinical complexity of this case is attributable not only to the severity of liver injury but also to its distinctive clinical phenotype, namely rare pseudo-progressive portal lymphadenopathy. Notably, although lymph node enlargement during EGFR-TKI therapy is generally interpreted as disease progression, direct reports of EGFR-TKI-associated reactive lymphadenopathy remain extremely limited. Nevertheless, increasing evidence suggests that EGFR-TKIs may induce immune-mediated inflammatory reactions involving multiple organ systems, including the liver and skin, direct reports of EGFR-TKI-associated reactive lymphadenopathy remain extremely limited. Nevertheless, increasing evidence suggests that EGFR-TKIs may induce immune-mediated inflammatory reactions involving multiple organ systems, including the liver and skin (Cheng et al., 2022; Uetrecht, 2019; Mak and Uetrecht, 2017). In this context, Kunimasa et al. reported a case of dermatopathic lymphadenopathy that occurred during osimertinib treatment. Initially, the newly enlarged lymph nodes were suspected to indicate disease progression. However, subsequent pathological evaluation confirmed that the lymphadenopathy was due to benign reactive changes rather than metastatic involvement (Kunimasa et al., 2020). Although the reported lymphadenopathy occurred in a dermatopathic rather than a hepatic setting, the case demonstrates that EGFR-TKI-induced immune reactions may result in benign reactive lymphadenopathy that closely mimics tumour progression on imaging. Unlike the case reported by Kunimasa et al., which was accompanied by prominent dermatological manifestations, our patient developed isolated portal lymphadenopathy without systemic hypersensitivity features. This made recognising this inflammatory process substantially more challenging. Therefore, the major diagnostic challenge in this present case was distinguishing reactive lymphadenopathy from genuine disease progression. In the present case, a range of alternative diagnoses were systematically excluded through comprehensive laboratory and imaging investigations. These diagnoses included viral hepatitis, autoimmune liver disease, biliary obstruction, choledocholithiasis, and hepatic metastasis (EASL Clinical Practice Guidelines, 2019; Chalasani et al., 2021). In view of the fact that DILI presents in a heterogeneous manner, it is imperative to differentiate it from hepatic metastasis and other hepatobiliary disorders (Hoofnagle and Björnsson, 2019). The diagnosis of furmonertinib-induced idiosyncratic DILI was substantiated by several lines of evidence. These included a compatible latency period following drug exposure, the exclusion of competing etiologies, and a RUCAM score of 9, indicating a highly probable causal relationship between furmonertinib and liver injury (Danan and Teschke, 2015; Uetrecht, 2019).

From a pharmacokinetic perspective, furmonertinib undergoes oxidative metabolism primarily via the hepatic cytochrome P450 system, particularly the CYP3A4 isoenzyme (Zhou Q. et al., 2024). One possible explanation is inter-individual variability in metabolic detoxification pathways, which may result in the accumulation of reactive metabolites, which could result in abnormal hepatic accumulation of the electrophilic reactive metabolites of furmonertinib (Roth and Lee, 2017). In accordance with the classical ‘Hapten Hypothesis’, these chemically active intermediates covalently bind to hepatic cellular proteins, forming ‘hapten-carrier complexes’. These complexes are subsequently recognised by the immune system as neoantigens, thereby activating CD8^+^ T Cells and inducing an immune-mediated hepatic cellular injury response (Uetrecht, 2019; Mak and Uetrecht, 2017). The present immune-related pathogenesis shares immunopathological similarities with previously reported liver injury associated with first-generation EGFR-TKIs, such as gefitinib, potentially explaining the immunomediated manifestations observed in this case.

It is noteworthy that the aforementioned immune-mediated injury did not cease clinically following drug withdrawal but instead progressed into a predominantly cholestatic pattern characterized by progressive hyperbilirubinemia despite declining transaminase levels. Although ALT declined rapidly with drug clearance, total bilirubin paradoxically increased within 1 week after discontinuation (203.5 → >280 μmol/L). This revealed that the pathological mechanism had shifted from simple cellular necrosis to functional cholestasis. Research indicates that inflammatory cytokines released following T-cell activation (particularly TNF-α and IL-6) can directly suppress the expression of bile salt export pump (BSEP/ABCB11) at the transcriptional level (Zollner and Trauner, 2008). This molecular-level ‘traffic jam’ paralyses bile excretion function, and conventional anti-inflammatory hepatoprotective drugs cannot reverse this transporter inhibition. This may explain why bilirubin levels only rapidly normalised following corticosteroid administration. Furthermore, the continued elevation of bilirubin after cessation of furmonertinib suggests that disease progression was no longer driven solely by direct drug toxicity but was likely maintained by ongoing immune activation. In contrast, glucocorticoids have been demonstrated to inhibit T-cell activation, decrease pro-inflammatory cytokine production, and diminish antigen-presenting cell function. This results in the interruption of the perpetuation of immune-mediated liver injury (Uetrecht, 2019; Mak and Uetrecht, 2017). The rapid decline in bilirubin levels and marked improvement in liver function following glucocorticoid therapy provide further evidence that immune-mediated mechanisms played a significant role in the ongoing progression of liver damage in this case. The concomitant regression of portal lymphadenopathy further suggests that both hepatic injury and nodal enlargement were driven by a common immune-inflammatory process. Importantly, newly developed isolated portal lymphadenopathy during EGFR-TKI therapy should not automatically be interpreted as disease progression, particularly when accompanied by severe hepatotoxicity and continued response of the primary tumour. Consequently, corticosteroid therapy may be considered in carefully selected patients with suspected immune-mediated idiosyncratic DILI who exhibit progressive cholestasis despite withdrawal of the offending agent and conventional supportive treatment. However, given the paucity of evidence supporting the use of glucocorticoids in DILI, treatment decisions should remain individualised and be made within a multidisciplinary clinical framework.

Anatomically, hepatic lymph drains directly through the Space of Disse to the first sentinel station—the hepatic lymph nodes (Ohtani and Ohtani, 2008). During severe hepatic parenchymal inflammation, large amounts of inflammatory mediators and antigen-presenting cells are transported through this pathway, leading to acute reactive lymphoid hyperplasia in the hepatic hilum. This mechanism provides a plausible pathophysiological explanation for the newly observed portal lymphadenopathy observed during the acute phase of liver injury in the present case.

The differentiation between reactive lymphadenopathy and metastatic lymph node involvement is critical in oncologic imaging. Reactive lymphadenopathy typically retains benign morphological characteristics, including preservation of nodal architecture, an oval configuration, and relatively homogeneous enhancement. In contrast, metastatic lymph nodes are more likely to exhibit suspicious features such as a rounded morphology, central necrosis, nodal conglomeration, heterogeneous enhancement, or progressive enlargement over time (Ahuja et al., 2008; Ying et al., 2014; Koh and Collins, 2007). In the present case, no central necrosis, nodal conglomeration, or other radiological features suggestive of metastatic disease were observed, and the onset of enlarged lymph nodes in the portal region was highly consistent in timing with the deterioration of liver function parameters and the worsening of jaundice. Concurrently, a subsequent reduction in the levels of the two proteins coincided with an improvement in clinical symptoms and laboratory parameters. This clear temporal correlation provides further support for the diagnosis of inflammation-driven reactive lymphadenopathy, rather than true tumour progression or metastasis.

Although histopathological examination remains the gold standard for distinguishing benign from malignant lymphadenopathy, invasive biopsy was not pursued in this case. In the MDT discussion, both CT-guided biopsy and EUS-FNA were evaluated. However, a safe access route could not be identified due to the proximity of the lymph node to the portal vein, hepatic artery, and biliary structures. Furthermore, the presence of severe DILI, accompanied by coagulation abnormalities, resulted in a considerable escalation in the risk of bleeding. In view of the ongoing reduction in the primary lung lesion and the absence of other evidence of disease progression, close radiological surveillance was deemed to be a more appropriate course of action than invasive tissue sampling.

Following glucocorticoid therapy, both liver biochemical abnormalities and portal lymphadenopathy exhibited rapid improvement. The synchronous identification of laboratory and imaging abnormalities provides evidence for the pivotal role of immune-mediated inflammation in both hepatic injury and lymph node enlargement. The complete resolution of portal lymphadenopathy during follow-up further reduces the likelihood of metastatic disease and supports a reactive inflammatory process rather than tumour progression. From a diagnostic perspective, this response can be considered a classic example of diagnosis ex juvantibus. Absent histopathological confirmation, the temporal association between steroid administration and simultaneous improvement in jaundice, liver function, and lymph node size provides indirect yet significant evidence of an immune-mediated mechanism. This case underscores a clinically under-recognized phenomenon in EGFR-TKI therapy: isolated portal lymphadenopathy associated with severe drug-induced liver injury may mimic disease progression and lead to potential misinterpretation as treatment resistance. It is imperative to be cognizant of this pattern to avert the premature discontinuation of effective targeted therapy.

To our knowledge, this is the first reported case of furmonertinib-associated severe drug-induced liver injury accompanied by reversible portal lymphadenopathy mimicking disease progression. Importantly, recognition of this phenomenon may prevent inappropriate classification of treatment resistance and avoid unnecessary discontinuation of otherwise effective EGFR-TKI therapy. This case expands the spectrum of EGFR-TKI-related immune-mediated toxicity and highlights a rare diagnostic pitfall in which isolated portal lymphadenopathy during targeted therapy may be misinterpreted as tumor progression or treatment resistance. Importantly, when lymphadenopathy occurs concurrently with severe drug-induced liver injury and comprehensive evaluation excludes infection, metastasis, and other alternative causes, clinicians should consider the possibility of inflammatory pseudo-progression. In such cases, multidisciplinary assessment and careful clinical correlation—including temporal changes in liver function, imaging findings, and treatment response—are essential to avoid premature discontinuation of effective targeted therapy. In selected patients with suspected immune-mediated injury, a carefully monitored trial of glucocorticoid therapy may provide both therapeutic benefit and additional diagnostic information based on treatment response.

## Conclusion

In summary, we present what is, to the best of our knowledge, the first documented case of furmonertinib-associated severe idiosyncratic DILI accompanied by reversible portal lymphadenopathy, mimicking disease progression. This case study serves to expand the existing body of knowledge on the spectrum of immune-mediated toxicities associated with EGFR-TKIs, and it underscores a noteworthy diagnostic challenge. Specifically, it highlights a rare instance in which inflammatory reactive lymphadenopathy may be misinterpreted as tumour progression or treatment resistance. Clinicians should be aware that the development of isolated portal lymphadenopathy during EGFR-TKI therapy, particularly when accompanied by severe liver injury and continued response of the primary tumour, does not necessarily indicate disease progression. In order to arrive at an accurate diagnosis, it is essential to integrate clinical presentation, dynamic biochemical changes, serial imaging findings and multidisciplinary assessment in a careful manner. Early recognition of immune-mediated liver injury and timely, individualised corticosteroid therapy in selected patients may facilitate recovery, reduce unnecessary invasive investigations, and help avoid premature discontinuation of otherwise effective targeted therapy.

## Data Availability

The original contributions presented in the study are included in the article/Supplementary Material, further inquiries can be directed to the corresponding authors.
