# Supplementary material for: Corticosteroid-responsive portal lymphadenopathy mimicking malignant progression induced by furmonertinib in EGFR-mutated NSCLC: a case report
Source: Front Pharmacol. 2026 Jul 23;17:1827175. doi: 10.3389/fphar.2026.1827175 (PMC13442828; doi:10.3389/fphar.2026.1827175)
Supplement: Supplementary file 1 [file Table1.docx]

| **Table S1. Updated RUCAM for the hepatocellular injury of DILI and HILI.** | | |
| --- | --- | --- |
| **Items for Hepatocellular Injury** | **Score** | **Result** |
| 1. Time to onset from the beginning of the drug/herb |  | 2 |
| ●   5–90 days (rechallenge: 1–15 days) | +2 | □ |
| ●   <5 or >90 days (rechallenge: >15 days) | +1 | □ |
| Alternative: Time to onset from cessation of the drug/herb |  |  |
| ●   ≤15 days (except for slowly metabolized chemicals: >15 days) | +1 | □ |
| 2. Course of ALT after cessation of the drug/herb |  | 3 |
| Percentage difference between ALT peak and N |  |  |
| ●   Decrease ≥ 50% within 8 days | +3 | □ |
| ●   Decrease ≥ 50% within 30 days | +2 | □ |
| ●   No information or continued drug use | 0 | □ |
| ●   Decrease ≥ 50% after the 30th day | 0 | □ |
| ●   Decrease < 50% after the 30th day or recurrent increase | −2 | □ |
| 3. Risk factors |  | 1 |
| ●   Alcohol use (current drinks/d: >2 for women, >3 for men) | +1 | □ |
| ●   Alcohol use (current drinks/d: ≤2 for women, ≤3 for men) | 0 | □ |
| ●   Age ≥ 55 years | +1 | □ |
| ●   Age < 55 years | 0 | □ |
| 4. Concomitant drug(s)/herb(s) |  | 0 |
| ●   None or no information | 0 | □ |
| ●   Concomitant drug/herb with incompatible time to onset | 0 | □ |
| ●   Concomitant drug/herb with compatible or suggestive time to onset | −1 | □ |
| ●   Concomitant drug/herb known as hepatotoxin and with compatible or suggestive time to onset delete marking right side above | −2 | □ |
| ●   Concomitant drug/herb with evidence for its role in this case (positive rechallenge or validated test) | −3 | □ |
| 5. Search for alternative causes | Tick if negative | Tick if not done |
| Group I (7 causes) |  |  |
| ●   HAV: Anti-HAV-IgM | □ | □ |
| ●   Hepatobiliary sonography / colour Doppler | □ | □ |
| ●   HCV: Anti-HCV, HCV-RNA | □ | □ |
| ●   HEV: Anti-HEV-IgM, anti-HEV-IgG, HEV-RNA | □ | □ |
| ●   Hepatobiliary sonography/colour Doppler sonography of liver vessels/ endosonography/CT/MRC | □ | □ |
| ●   Alcoholism (AST/ALT ≥ 2) | □ | □ |
| ●   Acute recent hypotension history (particularly if underlying heart disease) | □ | □ |
| Group II (5 causes) |  |  |
| ●   Complications of underlying disease(s) such as sepsis, metastatic malignancy, autoimmune hepatitis, chronic hepatitis B or C, primary biliary cholangitis or sclerosing cholangitis, genetic liver diseases | □ | □ |
| ●   Infection suggested by PCR and titer change for |  |  |
| ●  CMV (anti-CMV-IgM, anti-CMV-IgG) | □ | □ |
| ●  EBV (anti-EBV-IgM, anti-EBV-IgG) | □ | □ |
| ●  HSV (anti-HSV-IgM, anti-HSV-IgG) | □ | □ |
| ●  VZV (anti-VZV-IgM, anti-VZV-IgG) | □ | □ |
| Evaluation of groups I and II |  | 1 |
| ●   All causes-groups I and II—reasonably ruled out | +2 | □ |
| ●   The 7 causes of group I ruled out | +1 | □ |
| ●   6 or 5 causes of group I ruled out | 0 | □ |
| ●   Less than 5 causes of group I ruled out | -2 | □ |
| ●   Alternative cause highly probable | -3 | □ |
| 6. Previous hepatotoxicity of the drug/herb |  | 2 |
| ●   Reaction labelled in the product characteristics | +2 | □ |
| ●   Reaction published but unlabelled | +1 | □ |
| ●   Reaction unknown | 0 | □ |
| 7. Response to unintentional reexposure |  | 0 |
| ●   Doubling of ALT with the drug/herb alone, provided ALT below 5N before reexposure | +3 | □ |
| ●   Doubling of ALT with the drug(s)/herb(s) already given at the time of first reaction | +1 | □ |
| ●   Increase of ALT but less than N in the same conditions as for the first administration | −2 | □ |
| ●   Other situations | 0 | □ |
| Total score for the case | | 9 |

**Supplementary Table S1**

**Abbreviations:** ALT (1749 U/L), alanine aminotransferase; ALP (600 U/L), alkaline phosphatase; AST, aspartate aminotransferase; DILI, drug-induced liver injury; EGFR-TKI, epidermal growth factor receptor tyrosine kinase inhibitor; RUCAM, Roussel Uclaf Causality Assessment Method; TBIL, total bilirubin.

**Note:** The R-value was calculated at onset as (ALT/ULN) / (ALP/ULN), falling within the the hepatocellular injury criteria (R＞5). According to the updated RUCAM consensus guidelines, a total score of 6–8 signifies a "probable" relationship, while a score ≥ 9 confirms a "highly probable" etiology. The Negative Rechallenge in item 7 is strictly scored as 0 here because the subsequent re-exposure was conducted under optimized clinical monitoring/corticosteroid protection, which mathematically qualifies as an uninterpretable standard rechallenge under pure standalone drug conditions.
